# Supplementary material for: Ablation of Slc26a6 Mitigates Myocardial Ischemia/Reperfusion Injury
Source: Biomedicines. 2025 Nov 25;13(12):2874. doi: 10.3390/biomedicines13122874 (PMC12730430; doi:10.3390/biomedicines13122874)
Supplement: Supplementary file 1 [file biomedicines-13-02874-s001.zip › biomedicines-3938042-supplementary.pdf]

Supplemental Figures

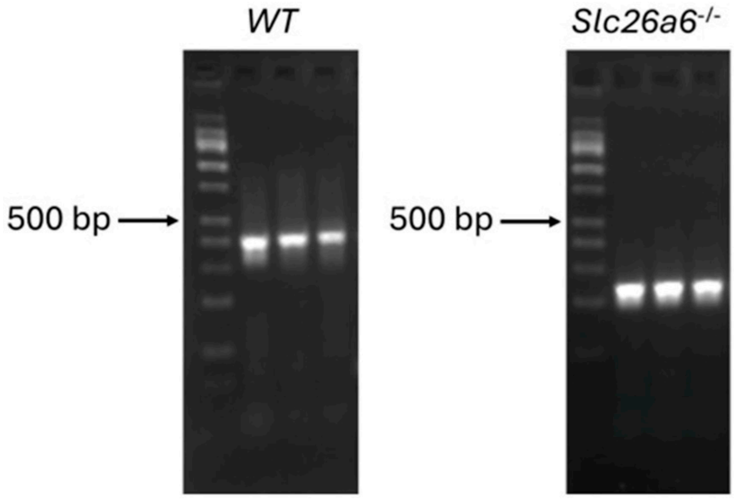

Figure S1. Photomicrograph showing genotype analyses using RT-PCR for wild-type (*WT*), and *Slc26a6*$^{-/-}$ mice. Each line represents the result from one mouse. The expected sizes for the 2 bands are 400bp and 250 bp for the WT and mutant alleles, respectively. Three sets of primers were used with 2 forward (F) primers for WT and mutant (KO) alleles and one reverse (R) primer for both WT and mutant alleles.

The primers used are listed as below:

Slc26a6-WT-F: CAAAGCCCTGGCTTCAGGGTGAATGATCTAG;

Slc26a6-KO-F: CTTCCATTGCTCAGCGGTGCTGTCCATCTG;

Slc26a6-WT-KO-R: GAAGAGGCCGACCAGGAAGCTGAGTGTGTAG.

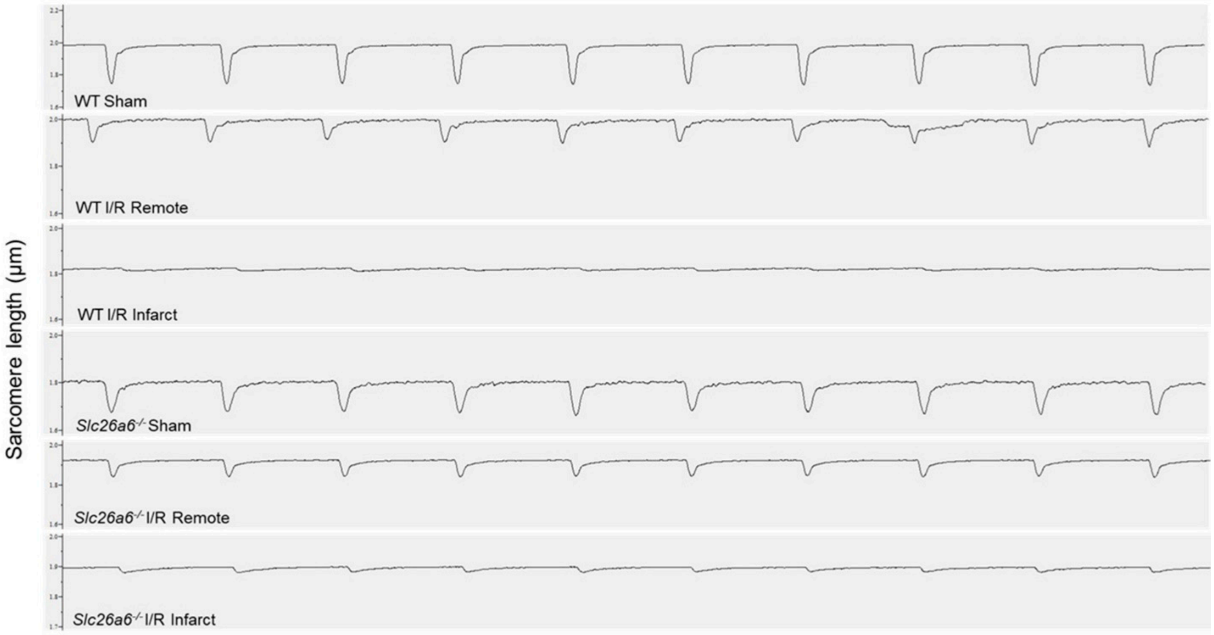

Figure S2. The original time-course of sarcomere shortening recorded in WT and *Slc26a6*$^{-/-}$ cardiomyocytes isolated from sham control, I/R remote and I/R infarct zone. The duration between two shortening peaks is 2 seconds.
